# Water, Forests, People: The Swedish Experience in Building Resilient Landscapes

**DOI:** 10.1007/s00267-018-1066-x

**Published:** 2018-05-21

**Authors:** Mats Eriksson, Lotta Samuelson, Linnéa Jägrud, Eskil Mattsson, Thorsten Celander, Anders Malmer, Klas Bengtsson, Olof Johansson, Nicolai Schaaf, Ola Svending, Anna Tengberg

**Affiliations:** 1 Stockholm International Water Institute (SIWI), Stockholm, Sweden; 2 Swedish Forest Agency, Jonkoping, Sweden; 3 Chalmers University of Technology, Gothenburg, Sweden; 4 Swedish University of Agricultural Sciences, Uppsala, Sweden; 5 SCC Forestry, Uppsala, Sweden; 6 Sveaskog AB, Stockholm, Sweden; 7 Stora Enso AB, Stockholm, Sweden

**Keywords:** Sustainable forest management, Integrated landscape approach, Forest institutions, Watershed management, Resilience, Landscape restoration, Swedish forest history

## Abstract

A growing world population and rapid expansion of cities increase the pressure on basic resources such as water, food and energy. To safeguard the provision of these resources, restoration and sustainable management of landscapes is pivotal, including sustainable forest and water management. Sustainable forest management includes forest conservation, restoration, forestry and agroforestry practices. Interlinkages between forests and water are fundamental to moderate water budgets, stabilize runoff, reduce erosion and improve biodiversity and water quality. Sweden has gained substantial experience in sustainable forest management in the past century. Through significant restoration efforts, a largely depleted Swedish forest has transformed into a well-managed production forest within a century, leading to sustainable economic growth through the provision of forest products. More recently, ecosystem services are also included in management decisions. Such a transformation depends on broad stakeholder dialog, combined with an enabling institutional and policy environment. Based on seminars and workshops with a wide range of key stakeholders managing Sweden’s forests and waters, this article draws lessons from the history of forest management in Sweden. These lessons are particularly relevant for countries in the Global South that currently experience similar challenges in forest and landscape management. The authors argue that an integrated landscape approach involving a broad array of sectors and stakeholders is needed to achieve sustainable forest and water management. Sustainable landscape management—integrating water, agriculture and forests—is imperative to achieving resilient socio-economic systems and landscapes.

## Introduction

Anthropogenic pressures on the Earth’s system have reached a scale at which significant global environmental change can no longer be avoided (Steffen et al. 2015). One dimension of this increased pressure is agricultural and urban expansion leading to forest degradation and loss of forest cover, which pose a considerable risk to unique biodiversity and habitats, and therefore are major global threats to ecosystems and humans alike. Of the original global forest cover in the world, about 15% has remained intact, 37% is fragmented, 20% is degraded and 28% is deforested (FAO 2015a; Laestadius et al. 2012).

Forests and trees also play a crucial role in the hydrological cycle (Bonan 2008; Livesley et al. 2016). They influence the amount of water available to humans and nature, and regulate the division between surface and groundwater flows, as well as interception and evapotranspiration. The negative effect of forest degradation and deforestation on water resources is not adequately understood or given due emphasis, despite the centrality of trees and forests to effective water resource management—particularly for countries in seasonally dry regions. Forests and trees also have great importance for the provision of other ecosystem services such as biodiversity, climate regulation and erosion control that increase social, economic and ecological resilience (Schroth and McNeely 2011; Wingfield et al. 2015; Kuyah et al. 2016; Reed et al. 2017). Therefore, sustainable management of the remaining forest, and restoration of degraded forests, is essential to safeguard important ecosystem services, and to secure long-term availability and sustainability of water resources (Stanturf et al. 2014).

This article analyzes Sweden’s experience with integrating forest and water management through multi-stakeholder participation. It highlights the urgent need for global landscape restoration and sustainable forest and landscape management. To this end, we suggest that experiences gained from the extensive forest restoration process that has taken place in Sweden over the last century can also be of relevance outside of Sweden.

The Swedish forest restoration experience and the identified supporting conditions can inform and enable new restoration initiatives globally to ensure the integration of a broader set of ecosystem services in landscape restoration. A possible entry point, building on the Swedish experience, is the “landscape approach”. This approach recognizes the need to be holistic both in time and space and to include all concerned stakeholders (Sayer et al. 2013; Freeman et al. 2015; Chazdon and Laestadius 2016; Reed et al. 2016). The Swedish context provides a valuable learning space as the successful restoration of Swedish forests would not have been possible without broad multi-stakeholder participation and integrated landscape management.

This paper provides a background on the interlinkages between water, forests and the larger landscape context in Swedish restoration efforts. After outlining the methodology on which this paper is based, the past and current Swedish experience in restoring the Swedish forest landscape is presented, as well as future challenges associated with participatory and integrated forest landscape management approaches. After a discussion of the challenges to landscape restoration worldwide, the final section concludes with a review of key factors of successful forest landscape restoration and the relevance of the Swedish experience for landscape restoration efforts elsewhere.

## Interlinkages between Water, Forests and the Landscape

Trees and forests in the landscape are central to managing water resources based on their influence on infiltration, evapotranspiration, surface runoff and sub-surface flows (Gómez-Baggethun and Barton 2013; Hansen et al. 2013; Bargués Tobella et al. 2014). In most cases, the presence of forests in the landscape provides both local and regional benefits that far outweigh the costs of reduced water flows in rivers due to the water use of trees (Bargués Tobella et al. 2014).

In contrast with sectorial approaches, the landscape approach aims to simultaneously address several of the global challenges that we are currently facing, such as the impact of extreme water hazards driven by climate change, food insecurity and poverty. It provides a framework for addressing the increasingly complex and widespread social, environmental and political drivers that typically transcend traditional management boundaries (Sayer et al. 2013; Reed et al. 2015, 2016). The landscape approach is multi-sectorial and brings together different actors from industry, local communities and government, to negotiate conservation and development trade-offs in the management of natural resources.

Of great and increasing importance are the positive impacts that a well-maintained forest or tree-covered landscape can have for downstream areas. This is particularly relevant given the current context of growing urbanization worldwide, with cities depending on the larger water basin in which they are located for supply of sufficient and good quality water as well as food and energy (Eriksson et al. 2014). Upstream–downstream linkages and interdependencies are therefore receiving increased attention from researchers and policymakers alike (Earle et al. 2015). This includes the important connection between the impact of upstream fresh water flows on downstream brackish and saline sea water in a source-to-sea perspective (Granit et al. 2016). However, since these interlinkages can be both positive and negative, it is important to disentangle the connections for evidence-based policy and decision making (Ilstedt et al. 2016).

A topical question is whether forests contribute to better water availability downstream, or whether they reduce the amount of water that can be withdrawn for societal purposes (Farley et al. 2005; Ilstedt et al. 2007; Grant et al. 2013). The question is particularly relevant in semi-arid environments where the outcome can significantly impact local communities. Traditionally, forests have often been described as “sponges”, storing and slowly releasing rainwater to maintain groundwater and streams during dry periods (Ilstedt et al. 2016). However, with increasing water demand, and with accumulating evidence from forest plantations, the positive role of forests in groundwater recharge has been much questioned in semi-arid settings (Farley et al. 2005). These empirics notably do not contain studies in the semi-arid tropics considering restoration of degraded land and more typical landscapes with partial forest cover and agroforestry (Malmer et al. 2010). Thus, depending on the climate regime and the particular area, water consumption by forest may or may not pose a problem (Sandström 1995; Ellison et al. 2011).

In many cases where a prominent monsoon climate leads to excessive water and subsequent flooding and inundation of large areas during a part of the year and drought situations during another, a reduced but more even water provision may be the preferred situation. In other cases where the annual precipitation is very low, it is of higher priority to ensure that as much water as possible is made available downstream. However, recent research (Ilstedt et al. 2016) suggests that there are possibilities to find an optimum tree density that facilitates groundwater recharge, where the benefit of even water provision outweighs the consumption of water by trees through transpiration. For instance, in regions where short intense and high magnitude rainfall events during the wet season can be highly destructive, triggering flash floods, mud flows and landslides, a well-managed forest cover can be highly important to reduce the impact of destructive rainfall and increase the resilience of natural resources and local communities (Blaikie and Brookfield 1987; Ola et al. 2015).

Water quality is also highly dependent on its surrounding environment. Several studies show that forests close to a watercourse play a significant role in improving water quality with positive impact on biodiversity (Bergquist 1999; Nyberg and Eriksson 2001). Factors such as temperature, pH, turbidity and nutrient load are all dependent on the surrounding forest. If these factors are favorable, the abundance and diversity of species inhabiting the water increases (Saunders et al. 2002). Also, other structures like dead woody debris, stones and organic material are of importance for the biology and heterogeneity of the watercourse (Bleckert et al. 2010). How forestry operations are conducted will therefore affect the biodiversity and the chemistry of the water (Hansen et al. 2013).

## Materials and Methods

This article was developed following a series of workshops and follow-up semi-structured discussions aiming to clarify and emphasize the interconnections between forests and water in general and particularly to identify key features in Swedish forestry practices and governance that contribute to maintaining the water provisioning services of forests (Samuelson et al. 2015). Professionals from the Swedish forest and water resource sector were invited to contribute. They represented forest authorities, universities and other research organizations, industry, consultancy companies, smallholder organizations and civil society organizations such as environmental and tree planting non-governmental organizations (NGOs) and the Swedish church (Table 1). Many of these organizations are involved in a variety of sustainable forest management support and development initiatives in low-income countries. Participants identified, analyzed and shared key components of successful water and forest resource management in Sweden. During three seminars spread over 1 year (2014–2015), 20 focus group discussions were held, each involving 5–10 participants. Key topics included: how forest and water management are related; what lessons can be learned from the Swedish experience; and what relevance these lessons have for global landscape restoration efforts and sustainable development. In total, more than 100 people from 42 academic, public and private sector organizations and NGOs participated in the process. Critical assessments of the inputs, findings and recommendations from these workshops and group discussions, and their linkages to existing literature, form the basis of this article.

**Table 1:** Agencies consulted in the process being part of fact finding for this article (*n* = 42)

Organization	Official website
Government Agencies	
Ministry of the Environment and Energy	www.government.se/government-of-sweden/ministry-of-the-environment/
Ministry of Enterprise and Innovation	www.government.se/government-of-sweden/ministry-of-enterprise-and-innovation/
Swedish Environmental Protection Agency	www.swedishepa.se/
Swedish Forest Agency	www.skogsstyrelsen.se/en/
Swedish International Development Cooperation Agency (SIDA)	www.sida.se/English
Swedish Meteorological and Hydrological Institute (SMHI)	www.smhi.se/en
Swedish Museum of Natural History	www.nrm.se/english
International organizations	
Food and Agriculture Organization of the United Nations, FAO	www.fao.org
Forest Trends	www.forest-trends.org
Certification organization	
Forest Stewardship Council (FSC) Sweden	https://se.fsc.org
Programme for the Endorsement of Forest Certification (PEFC)	http://pefc.org/
NGOs/charities	
CDP (Carbon Disclosure Project)	www.cdp.net
Stockholm International Water Institute (SIWI)	www.siwi.org
Swedish Society for Nature Conservation	www.naturskyddsforeningen.se/in-english/
Vi Agroforestry	www.viagroforestry.org/
World Wide Fund for Nature (WWF) International	www.org
Religious organizations	
Church of Sweden	www.svenskakyrkan.se/english
Universities/Research organizations	
Centre for International Forestry Research (CIFOR)	www.cifor.org
Forest, Climate and Livelihood Research Network at Chalmers University of Technology (Focali)	www.focali.se
Forest, Climate and Livelihood Research Network at Chalmers University of Technology (Focali)	www.focali.se
Gothenburg University	www.gu.se/english
IVL Swedish Environmental Research Institute	www.ivl.se
KTH Royal Institute of Technology	www.kth.se/en
Linköping University	www.liu.se/?l = en
Secretariat for International Forestry Issues (SIFI)	http://www.sifi.se/
Stockholm Environment Institute (SEI)	https://sei-international.org/
Stockholm Resilience Centre (SRC)	www.stockholmresilience.org/
Skogforsk (the Forestry Research Institute of Sweden)	www.skogforsk.se/english/
Swedish University of Agricultural Sciences (SLU), Global Programme	www.slu.se/en/
World Resources Institute	www.wri.org/
Producers/businesses	
Ekebo Forest Management AB	
Hamra Gård Consultancy	
LRF Forestry (The Federation of Swedish Family Forest Owners)	www.lrf.se/om-lrf/in-english/
NIRAS Consulting company	www.niras.se/
Nkoola Agencies International Ltd	http://nailug.com/home
Sense Group AB	
SSC Forestry	http://ssc-forestry.com/
Stora Enso AB	www.storaenso.com/
Swedish Forest Industries Federation	www.forestindustries.se/
Sveaskog AB	www.sveaskog.se/en/
Södra (Sodra)	www.sodra.com/en/
TetraPak AB	www.tetrapak.com/

## Results

### Swedish Forest Restoration in Historical Perspective

Sweden’s forest cover amounts to 28.1 million ha, or about 68% of its total land area (based on the FAO forest definition; FAO 2015a). Productive forestland, i.e. land that is suitable and intended for forest production, accounts for 23.2 million ha (82.6% of forest land) (Swedish Forest Agency 2014). Sweden also has abundant water resources, with a total freshwater withdrawal of only 1.5% of annual renewable water resources (FAO 2014).

The current forest ownership structure reflects the intention to privatize forest land that dates back over 200 years. The privatization process was initiated in the late seventeenth century, before industrialization, but gathered momentum with the adoption of a new law (*Laga skifte*) in 1827, through which former Crown forest land was distributed among smallholders (Ingemarson and Nylund 2013). This led to increased commercial value of the forest since it opened markets beyond limited homestead use and the interplay between the State, farmers and private companies gained importance (Ingemarson and Nylund 2013). At that time the State did not pay attention to user rights relating to historical or actual land use.

During the second half of the nineteenth century, steam-powered sawmilling and river transport made it possible to extract and process timber at a much higher rate (Josefsson and Östlund 2011). By the end of the 1800s and early 1900s, large parts of Sweden’s forest resources were deforested and degraded and reached its minimum extent due to a combination of industrialization, a growing population and the accompanying need for firewood and land for cultivation and grazing (Kardell 2004; Laestadius 2015). Parts of southern Sweden had become more or less devoid of forests, mainly as a result of the growing population needing additional areas for agriculture, while in the north, repeated selective logging to meet the demand from the growing industry had resulted in poorly stocked forests with insufficient regeneration (Ingemarson and Nylund 2013; Axelsson 2014).

One hundred years later, a significant restoration effort led to a complete change, with larger areas of managed forests than ever before, roughly a doubling of the total standing volume, and a highly developed forestry industry (Laestadius 2015). Once the subsistence economy had been replaced by a market economy, the transition to exclusive forest ownership occurred rapidly. A significant factor behind this achievement was the allocation of forest land to rural poor farmers during the nineteenth century combined with the demand for raw material for the expanding wood-based industries. Eventually around 250,000 homesteads were allocated 10 million hectares of forest with legal title to their land (Ingemarson and Nylund 2013), roughly corresponding to one-third of today’s total forest land in Sweden, or half of the productive forest land. It made sense to the government to manage forests for the benefits and income they could provide also for the rural poor, and government interventions protected the rural poor from being exploited by forest companies. A growing class of land-owning farmers contributed to political stability in a period during which the number of rural landless increased and urban industries could not absorb the labor excess. Ingemarson and Nylund (2013, p 6) conclude that the “government policy had achieved two goals: one of fiscal consolidation by increasing the number of taxpayers, and the other of securing political stability”. It is clear that private forest ownership in combination with efficient legislation contributed to the success of the Swedish forestry model, which largely builds on the economic dimension as a way to achieve sustainability and sustainable development (Beland Lindahl et al. 2017). It relies on fundamentals such as “stable institutions, markets and clear rules for the actors based on a democratic system” (Ingemarson and Nylund 2013, p 56).

**Fig. 1:**
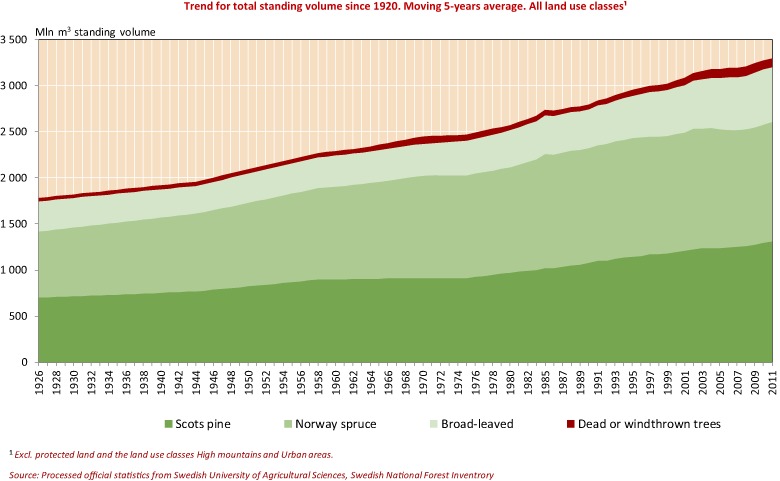
Trend for total standing volume in Swedish forests since 1920s (moving 5-year average). All land-use classes except protected land, high mountains and urban areas are presented. Adapted from the Swedish Forest Agency (2014)

When forest restoration in Sweden started in the late nineteenth century, it primarily focused on sustaining yields, improving forest management and providing industry with timber and pulpwood (Ingemarson and Nylund 2013). Only since the latter half of the twentieth century have other considerations, such as water resource management, environmental and social values, been increasingly integrated in management strategies and national forestry policies (KSLA 2015). From a production point of view, the Swedish case can be seen as an example of successful restoration of a national forest resource (Fig. 1). Below follows an elaboration of the key conditions that enabled the massive landscape restoration effort, the key features of Sweden’s integrated forest and water management, the challenges encountered and the lessons learned.

### Key Factors which Enabled the Gradual Swedish Forest Landscape Restoration

The transition in forest ownership throughout Sweden and the subsequent change from a depleted to a remarkably productive forest landscape took place over several hundred years (Kardell 2004). This section elaborates on the conditions that enabled this transition. Based on the workshop outcomes, we have identified five key factors that contributed to the successful Swedish forest restoration experience, which are further elaborated below.

#### Forest tenure and ownership

**Fig. 2:**
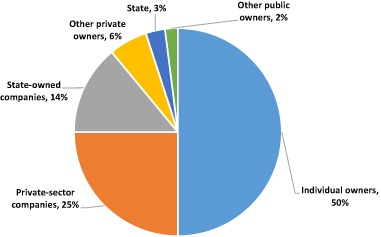
Productive forest land by owners classes in 2012. Adapted from Swedish Forest Agency (2014)

Private forest ownership and tenure have been important features of Swedish forest governance for at least 200 years when the privatization process gained momentum (Nylund 2009; Ingemarson and Nylund 2013). Approximately 81% of productive forest land in Sweden is now privately owned, with around 60% of the private owners comprised of smallholders, and 40% being forestry companies (Fig. 2). The number of smallholder estates has grown since the commencement of allocations in the early 1800s and today amounts to around 230,000 (including estates larger than 5 hectares), whereas the number of owners amounts to about 330,000 as some estates have more than one owner (a situation that occurs when holdings are passed from one generation to the next) (Swedish Forest Agency 2014). In the past, secure smallholder tenure paved the way for forest restoration efforts. The original distrust against the government, much linked to oak trees being the property of the government, was gradually turned into trust that investments in their land could be kept (Laestadius 2015). Over time, owners increasingly started trusting that their investments in regeneration and sustainable forest management would generate future financial return for their families. This has enabled the creation and development of well-organized forest owner associations, as well as competitive forest companies. In line with Hanson et al. (2015), we conclude that clear ownership of land and/or ensuring that local people can benefit from investments is key to successful forest restoration.

#### Legislation, governance and clear rules

The evolution toward the present forestry governance arrangements began around 1900. In 1903, a new Forestry Act prescribed regeneration of forests owned and harvested by private forest owners. It was a framework with relatively vague prescriptions, but nevertheless is believed to have brought success through counseling, education and persuasion (Nylund 2009; Appelstrand 2012). Transparent systems and the avoidance of corruption have been key to progressive forestry and forest industry development in Sweden and hence clear rules and “good governance” have been important key factors for successful forest restoration in Sweden. They are instrumental for securing an enabling institutional environment that strives to respect, protect and balance the rights of different actors, not least private forest owners (Disch et al. 2009). Furthermore, they have made it possible for both smallholders and companies to invest in forestry. Transparent regulatory frameworks, recognized user/owner rights, clearly marked holding boundaries and a functioning and fair wood market are some of the key features of the Swedish forest governance system (KSLA 2015). Another important feature is the national system for wood measurement (Swedish Wood 2016). Timber grading in Sweden is primarily carried out by independent regional timber measurement councils (The Timber Measurement Council 1999). Each council ensures that measurement work is carried out in a neutral and uniform way by qualified people employed by the council, independent of sellers and buyers. This system guarantees impartial assessment and accurate prices in the supply chain.

In 1993, a major revision of the Swedish Forestry Act (SFA) was undertaken leading to two major changes (Swedish Forestry Act 1993). First, an ambitious environmental goal was included in the SFA that led to environmental concerns being given equal value to the previous production goal. Secondly, the forest governance system was softened, replacing detailed regulation, command and control, monitoring and enforcement with information sharing and education and advisory services (Appelstrand 2012; Beland Lindahl et al. 2017). Forest owners were thereby given greater autonomy but were still required to take special measures to protect valuable biotopes, aquatic systems and cultural heritage. The expression “freedom under responsibility” became a signature of this change in governance system and characterizes “the Swedish Forestry Model” (KSLA 2009). There are differing perceptions of the effectiveness of the “freedom under responsibility” principle. Within the private sector it is considered successful, while many NGOs consider the progress toward environmental and social objectives to be slow and inadequate (Berglund 2014).

#### Public participation, education and capacity building

**Fig. 3:**
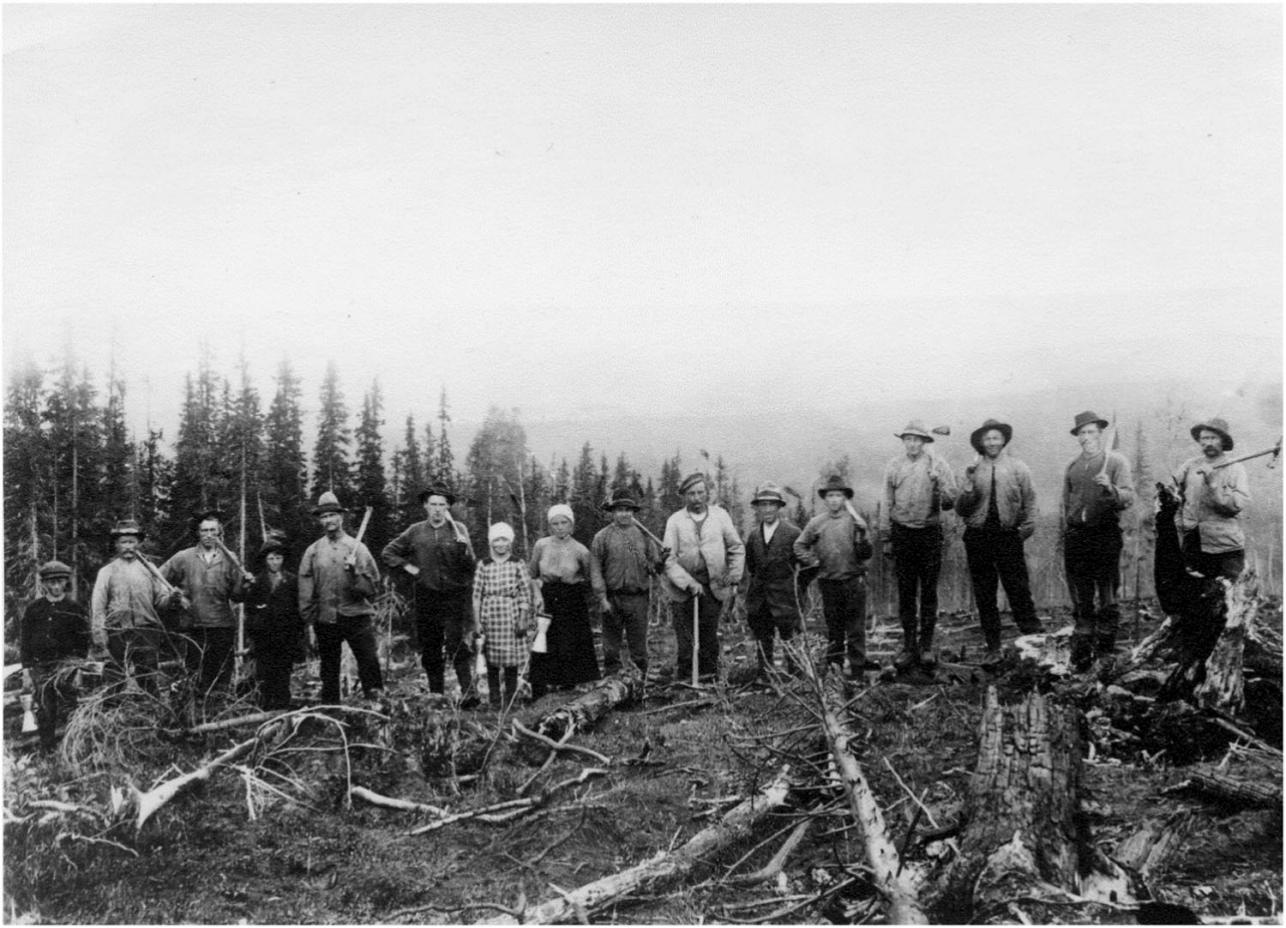
Small-holder land owners and their children replanting a clear-cut area in 1903, Storuman, Sweden. Source: Swedish University of Agricultural Sciences Archive

From the very start of the Swedish restoration transition, public awareness raising, advisory services and training in forest management to comply with legislation have been important tools, along with subsidies and law enforcement. These activities were initially carried out by the County Forestry Boards (CFBs) established by the government after the adoption of the first Forestry Act in 1903, and were later supported by other actors (Appelstrand 2012). Several civil society organizations supporting rural development and forest restoration became deeply involved and school classes were widely mobilized to help in planting activities (Fig. 3). The CFBs eventually evolved into the current Swedish Forest Agency. During the last century, the Swedish Forest Agency has repeatedly held major information campaigns with the aim being to train the landowners and those working in the forestry industry (KSLA 2015). Training has been important given the large number of landowners and the successive changes in legislation in later decades. The main message has varied over time: improved replanting prevailing in the 1920s to 1930s; enhanced timber production in the 1970s; consideration to nature values in the 1990s; and paying attention to the forests’ water system and recreational values since the 2000s. Training programs are ongoing and remain one of the main tasks of the Swedish Forest Agency. During the last decade, the Swedish Forest Farmers Associations have increasingly and successfully taken part in these public awareness-raising and education efforts (Laestadius 2015).

#### Integration of science and practice

The integration of science and practice started early in Sweden and has been instrumental for forestry development ever since (Laestadius 2015). In this regard the development of forestry during the 1900s was to a considerable extent guided by science and technology. The Swedish National Forest Inventory, established in the 1920s, provided the necessary knowledge of national forest resources. At the same time, long-term research and trials were carried out, e.g. on stand dynamics, seed and tree improvement, soils and nutrition. Further scientific attention was paid to mechanization, forest planning, logistics, forest health, fire prevention, weeding and soil preparation. Training and education campaigns were pivotal in the implementation of best available management practices (Ingemarson and Nylund 2013). It was considered important to increase the education levels of forest technicians and to establish a long-term plan for the capacity building of forest owners through the regional forestry agencies.

#### A prosperous forestry industry

The forestry industry has been an important driver for restoration of the Swedish forest landscape (Ingemarson and Nylund 2013). At an early stage, logging in northern Sweden generated significant profits that were used to restore deforested and degraded forest lands in southern Sweden (Laestadius 2015). Over time, demand for timber increased and provided economic incentives for ensuring sustained yields. In 2016, Swedish forestry industry exports were valued at USD 125 billion (The Swedish Forest Industries 2018). Of Sweden’s total industrial employment, exports, sales and added value, the forestry industry accounts for 11–13%. It is strongly export-oriented, and since the raw materials are mainly domestic and the import of forestry products is relatively small, the industry provides a significant contribution to Sweden’s trade balance. More than 85% of Sweden’s pulp and paper production is exported, and the corresponding figure for sawnwood products is over 70% (The Swedish Forest Industries 2018). The standing volume in Swedish forests has more or less doubled during the twentieth century, despite a doubling of the population, a continuous increase in harvest levels and increased allocation of forest land for protection (Fig. 1). Thus, although a thriving forest industry is also dependent on the previous key factors, a functional industry can also in itself be a key success factor in forest landscape restoration.

The components listed above remain important cornerstones of Swedish forest governance and management today and enable a continuous improvement in forest management and integration of water resource management.

### Forest and Water Integration for Landscape Restoration

The first forest restoration phase in Sweden was partly triggered by the Forest Act introduced in 1903 (Appelstrand 2012). Sweden has lately entered a second restoration phase, partly linked to the revised Swedish Forest Act introduced in 1993 that addresses the interlinked challenges of preserving and developing multiple ecosystem services, such as wood production, climate change mitigation and adaptation, biodiversity and recreational values, simultaneously on the same unit of land (Nylund 2010). The integration of water resources in forest management is one of these challenges.

The new approach protects forest diversity in designated forest areas as well as through general environmental considerations in day-to-day management. This approach is particularly important for preserving and improving water quality in small streams, rivers, lakes and mires. The current management model builds on an integrated approach, where conservation measures are incorporated into the production landscape. This is true for Sweden, but is also increasingly becoming an emerging worldwide trend (FAO 2015b).

Attention to integrated forest and water management also fits the EU Water Framework Directive, adopted in 2000, which required Swedish forest stakeholders to strengthen consideration to water resources in Swedish forestry practices (Berglund 2014). Several private and public initiatives have been launched since. Water availability and water-related disasters are not an issue in most parts of Sweden, so the main focus of these initiatives is water quality and conserving biodiversity. One example is the comprehensive strategy for “Water Landscapes” developed by Sveaskog, the state-owned timber trade company. These water landscapes are carefully selected forested watersheds, where consideration to water resources is the guiding principle when planning for production and conservation set-asides. The strategy aims at contributing to fulfilling the elements of the EU Water Directive and to achieve the goals of the Swedish Environmental Objectives. Management strategies include increased amounts of dead wood for improving aquatic environments, creation of new wetlands and establishing new spawning beds for fish and mussels.

The Blue Targeting planning tool (Box 1) is another example of integrated forest and water management (Lestander et al. 2015). Blue Targeting is a supportive checklist that helps forest planning and operations to ensure a riparian buffer zone wide enough to secure provision of forest ecosystem services. A simple protocol (digital or analog) has been developed to assess conservation values, impact and sensitivity of streams. This results in a classification of the water that targets the surrounding forest. It suggests the creation of buffer zones needed to protect and enhance the water biology in areas where forestry operations are conducted. This planning tool was developed in the early 2000s as a collaborative effort between academia, the World Wide Fund for Nature and the Swedish Forest Farmer Associations. This water planning tool for riparian forests is integrated in forest management planning, for which other tools based on remote sensing and field surveys are used. In these plans, each stand is documented and classified with information required for making forest management decisions (KSLA 2015). Stand descriptions normally include long-term goals, as well as production and environmental aspects. This information can be digitalized, and forestry planning today is normally carried out on a laptop computer, with all information available and updated online.

### Toward Greater Stakeholder Involvement

The second wave of integrated forest landscape restoration in Sweden is also characterized by extensive stakeholder dialogs as well as training and capacity building of the key actors (forest owners, entrepreneurs, foresters etc.), often facilitated by the Forest Agency. This multi-stakeholder approach has shaped the practical implementation of the current Forestry Act of 1993 (Jägrud 2007).

One example of an integrated approach with a broad participation of forest stakeholders is the Dialogue Project that was implemented in Sweden from 2011 to 2014. Responsible authorities—the Swedish Forest Agency and the Swedish Environmental Protection Agency—acknowledged the need for an extensive discussion on how to improve environmental values in the landscape (Berglund 2014). In this project, transdisciplinary groups were formed to establish environmental targets for water and soil, historical/cultural heritage structures as well as social and environmental values in the forest landscape. These groups met regularly over a period of 2 years to agree on appropriate environmental targets. Such targets were set for streams and lake buffer zones and stream passages, including the width and structure of a functional buffer zone next to a water course, and the distance between a water course and a production forest. These targets are now being implemented in the wider productive forest landscape, and not only specifically in areas set aside for environmental concerns (Andersson et al. 2013).

Another successful example of an integrated participatory approach is the training program “Water in Forests”, initiated by the Forest Owners Associations and the World Wide Fund for Nature (WWF). These organizations developed and implemented a training program for forest owners to enhance their awareness and consideration of water management in forestry operations. Once developed, the training program has become common practice among private forest owners association throughout Sweden (Bleckert et al. 2010). The “Blue Targeting” planning tool, elaborated in the previous section, was also implemented during this information campaign.

#### Box 1 Blue Targeting – A Tool for Water and Forest Management

The “Blue Targeting” is a tool to support sustainable water management and environmental conservation in the forest sector in Sweden (Lestander et al. 2015). The tool consists of a guided survey—(the Conservation values; Impact from humans; Sensitivity of soil; Added value (CISA) field sheet)—of binary (yes/no) questions (parameters) and a score system with answers based on observations in the field. The results of the survey are tallied to provide recommendations on appropriate riparian buffer zone management that will sustain healthy riparian ecosystems, including water quality. The tool will guide the needs for action in forest management, and gives good information about the water status (Ingemarsson 2012). The tool was developed by the Swedish Forest Owner association in collaboration with WWF and has now been successfully tested over several years (Ingemarsson 2012; Lestander et al. 2015). The CISA field sheet is divided into four sections: (a) conservation values (including the stream, special biotopes, riparian zone); (b) impact from humans (including hydro-morphological impact such as draining, straightening); (c) sensitivity of soil (including topography, soils in risk of erosion); and (d) added value (such as recreation, food production, cultural, actions for restoration). The result from the CISA field sheet exercise shows the water situation, which is then transformed into a classification that acts as indicator on what actions are recommended in forest management. The Blue Targeting tool is currently being adapted for other forest conditions outside Sweden. This work is undertaken within the EU Interreg Baltic Sea Region project on Water Management in Baltic Forests and the FAO Forest and Water Monitoring Framework, and is a good example of how Swedish forest management experiences can also support the forest sector in other countries.

## Discussion

Challenges such as a growing population, a growing middle class, a globalized market, water scarcity, environmental concerns and climate change will have profound impacts on future forests and forestry practices. While increased temperatures and longer growing seasons might be beneficial in some parts of the world such as in the boreal areas, they might be detrimental in other parts such as in low latitude areas. In arid and semi-arid parts of the world, in particular, changes in precipitation and increased evaporation may lead to lower productivity. Low latitude areas are also where most developing countries are located, and they may be less equipped to adjust and adapt to negative impacts of climate change. In general, the combination of challenges is likely to lead to an increased need globally for a more holistic approach to sustainable forest landscape management, improved management of natural resources, such as water, soils and ecosystems, and restoration of the vast areas of degraded forest land (Laestadius et al. 2012).

However, forest landscape restoration is complex (Chazdon 2008; Stanturf 2016). This is partly due to the broad spectrum of objectives, such as sustainable intensification of agricultural production, supporting improved and resilient livelihoods and promoting equitable value chains, which may stand in contrast to the desire for intensified forestry, urban expansion or increased protection of ecosystem values. Furthermore, context-specific nuances will influence outcomes as there will be different baselines related to local specifics of ecology, culture, degree of poverty, infrastructure and resource pressure in each targeted landscape (Freeman et al. 2015). Each intervention requires a cross-sectorial and transdisciplinary approach. Furthermore, policy and institutions at both local and national levels need to be strengthened in parallel. Stakeholder-owned dialogs constitute an important first step.

The rapid breakthrough for forest certification in Sweden illustrates how structural components in place can enable sustainable restoration practices (Nylund 2010). Forest certification was introduced in the late 1990s through the Forest Stewardship Council (FSC) and the Program for the Endorsement of Forest Certification (PEFC). More than half of productive forest is today certified by either FSC or PEFC. The certification systems add additional environmental and social considerations to government rules and regulations, such as the integration of water resource management in sustainable forestry (PEFC Sweden 2012; FSC Sweden 2017).

The current Swedish Forestry Act of 1993 provides a significant degree of freedom, but also responsibility for the forest owners to take action to achieve the objectives of the act (Lämås and Fries 1995; Nylund 2009, 2010; Appelstrand 2012; Beland Lindahl et al. 2017). This is a significant difference compared to the previous Act that was characterized by detailed regulations. There are exceptions, however, the most significant being related to the obligation to reforest after clear-cutting. Furthermore, the Act aims to balance production goals with environmental goals (including social and cultural ones). This has been a challenging task for the forestry stakeholders (e.g. smallholders, forestry industry, academia, civil society organizations and environmental NGOs, religious and indigenous groups and state authorities). There remain, despite the recent stakeholder dialogs and management developments, unresolved questions when it comes to details on how to balance these aspects. A continued inclusive governance model to strengthen trust between the wide spectra of forest and forestry stakeholders is seen as significant to find robust solutions for the future. Table 2 lists a selection of Swedish key stakeholders and their respective competences of relevance in a coordinated multi-sector framework for the sustainable management of resilient forest and tree landscapes.

**Table 2:** Swedish competences of special relevance for restoring sustainable forest landscapes, identified in discussions with the government agencies listed in Table 1

Competence	Indicative key stakeholder
1. Governance	
Broad stakeholder participation in development and implementation of forest policies	Ministry of Rural Affairs, Ministry of Environment and Energy, Ministry of Enterprise and Innovation, forest and environment government agencies, water authorities, forestry industry, forest owner associations, universities of natural and social science, civil and environmental NGOs, religious and indigenous groups
Production and environmental objectives in Swedish Forest Policy	Ministry of Rural Affairs, Ministry of Environment and Energy, Ministry of Enterprise and Innovation, forest and environment government agencies, water authorities
Linking science with practice	Universities of natural and social science, technological institutes, forest smallholders, associations, forestry industries, forest and environment government agencies, water authorities, civil and environmental NGOs, religious and indigenous groups
Capacity building in policy development and development of best management practices	Forest and environment government agencies, universities and scientific institutions, forestry industries, forest farmers associations, civil society and environmental NGOs, religious and indigenous groups
2. A prosperous forestry industry	
Product development and marketing of wood products	Forest technology and processing companies and entrepreneurs, trade associations, universities and knowledge institutions, designers, trade and investment councils
Cost-efficient and safe logging systems adapted for industry and small-scale businesses	Work environment authorities, forest technology entrepreneurs, forestry research institutes
Technology for competitive small- and medium-size mechanical wood industries	Forest technology entrepreneurs and industries, forest smallholder associations, designers
Technical tools and information technology (IT) solutions for forest management including inventories, maps, GIS and different information and communication technology solutions	Forest technology and IT entrepreneurs, technical social and natural science universities and academia, forestry research institutes
3. Prosperous forest smallholders	
Organization and empowerment of forest smallholders	Forest owner associations, certification schemes, trade unions
Entrepreneurship and business management including marketing, sale and export	Forest owner associations, trade associations, forestry entrepreneurs, universities and knowledge institutions, trade and investment councils
Fair and transparent systems for wood measurement and for making payments to smallholders	Wood measurement associations, forestry information hubs
Secure access to markets	Forest owner associations, trade and investment councils, certification schemes
4. Integrating water management in sustainable forestry	
Combined objectives (production, social and environmental) in forest management plans	Forestry companies and smallholders, supervised by forest and environment agencies and water authorities
Forest certification and chain of custody certification, including group certification of smallholders	Forestry companies, smallholders, certification schemes
Training in best management practices to forest owners (forestry companies and smallholders) forest supervisors, forest entrepreneurs and forest workers	Forest and environment agencies, water authorities, forest owner associations, universities and knowledge institutions (natural, technological, social)

The list of key stakeholders is not comprehensive. It is compiled by the authors as an indicator of the diversity of stakeholders needed for successful restoration and management of sustainable forest landscapes

Forest and landscape restoration will continue to be both a major challenge and an opportunity. In Sweden, attention is increasingly being paid to restoration of a wider range of environmental and social values. Meeting concomitant challenges will be a question of defining goals, direction and best practices, while acknowledging some of the key features that enabled the restoration of Swedish forest in the 20^th^ century. At the global level, some of the key structural features of forest restoration described in this article still need to be developed.

As the Swedish experience has shown, the challenge is to develop a coordinated approach that brings different actors and sectors together from forestry, agriculture, water resources, etc. The desired result would be a pooling of expertise for creating an enabling environment in terms of policies and institutions, and private sector engagement in sustainable forest management, including both small-scale forest owners and the large-scale forestry industry.

## Conclusions

Swedish forests have been successfully restored during the last hundred years, building a thriving natural resource base in a landscape largely depleted of forest. Forest policies and management strategies were initially focused on production but have lately started to integrate values such as climate change mitigation and adaptation, biodiversity conservation, social aspects and water resource management.

From the semi-structured discussions in the consultative process, involving a wide range of experts and stakeholders from various parts of the Swedish forest sector, key factors were identified for successful forest and landscape restoration in Sweden. These are (1) clear ownership of forest land; (2) clear rules and transparent decision making, avoiding corruption; (3) public participation in policy development, e.g., through forest farmers owners’ associations, awareness raising and capacity building; (4) integration of science with practice; and (5) the building of a prosperous forestry industry. These components continue to be important for integrated and multi-stakeholder governance and management of Swedish forest and water resources in productive landscapes and may well provide entry points for joint learning with developing countries and emerging economies.

Through development cooperation, Sweden’s experiences with landscape governance and integrated forest and water management could contribute to multilateral processes such as the global Bonn Challenge to restore 150 million hectares of deforested and degraded land by 2020 and 350 million by 2030, the New York Declaration (Climate Summit 2014), and the Governor’s Climate and Forest Task Force, all of which set targets and build on country commitments for restoration of degraded forest landscapes. Effective landscape restoration processes and better integration of ecosystem services in landscape restoration for the benefit of people, forests and water contributes to resilient landscapes that are key to sustainable development.
